# INTRAGO: intraoperative radiotherapy in glioblastoma multiforme – a Phase I/II dose escalation study

**DOI:** 10.1186/1471-2407-14-992

**Published:** 2014-12-22

**Authors:** Frank A Giordano, Stefanie Brehmer, Yasser Abo-Madyan, Grit Welzel, Elena Sperk, Anke Keller, Frank Schneider, Sven Clausen, Carsten Herskind, Peter Schmiedek, Frederik Wenz

**Affiliations:** Department of Radiation Oncology, University Medical Center Mannheim, University of Heidelberg, Theodor-Kutzer-Ufer 1-3, 68167 Mannheim, Germany; Department of Neurosurgery, University Medical Center Mannheim, University of Heidelberg, Mannheim, Germany; Department of Clinical Oncology and Nuclear Medicine (NEMROCK), Cairo University, Cairo, Egypt

## Abstract

**Background:**

Glioblastoma multiforme (GBM) is the most frequent primary malignant brain tumor in adults. Despite multimodal therapies, almost all GBM recur within a narrow margin around the initial resected lesion. Thus, novel therapeutic intensification strategies must target both, the population of dispersed tumor cells around the cavity and the postoperative microenvironment. Intraoperative radiotherapy (IORT) is a pragmatic and effective approach to sterilize the margins from persistent tumor cells, abrogate post-injury proliferative stimuli and to bridge the therapeutic gap between surgery and radiochemotherapy. Therefore, we have set up INTRAGO, a phase I/II dose-escalation study to evaluate the safety and tolerability of IORT added to standard therapy in newly diagnosed GBM. In contrast to previous approaches, the study involves the application of isotropic low-energy (kV) x-rays delivered by spherical applicators, providing optimal irradiation properties to the resection cavity.

**Methods/Design:**

INTRAGO includes patients aged 50 years or older with a Karnofsky performance status of at least 50% and a histologically confirmed (frozen sections) supratentorial GBM. Safety and tolerability (i.e., the maximum tolerated dose, MTD) will be assessed using a classical 3 + 3 dose-escalation design. Dose-limiting toxicities (DLT) are wound healing deficits or infections requiring surgical intervention, IORT-related cerebral bleeding or ischemia, symptomatic brain necrosis requiring surgical intervention and early termination of external beam radiotherapy (before the envisaged dose of 60 Gy) due to radiotoxicity. Secondary end points are progression-free and overall survival.

**Trial registration:**

The study is registered with clinicaltrials.gov, number: NCT02104882 (Registration Date: 03/26/2014).

## Background

Despite recent advances in therapy, Glioblastoma multiforme (GBM) is a lethal disease in most cases with a relatively short overall survival of roughly 15 months [1, 2]. In virtually all cases, GBM recur locally within a narrow margin (2–3 cm) around the tumor cavity [3–5]. Although GBM are highly invasive and able to migrate along pre-existing structures such as blood vessels or white matter tracts [6, 7] most (if not all) recurrent tumors paradoxically grow in close proximity to the resection margin [8]. Thus, though novel surgical techniques (such as fluorescence-guided resection) may have improved the rates of macroscopic complete resections [9], and advanced radiotherapy techniques are at hand, no single or combined approach is sufficient to deplete microscopically dispersed tumor cells around the tumor cavity.

One of the techniques employed to tackle this challenging feature of GBM is intraoperative radiotherapy (IORT). IORT allows the delivery of high doses of electrons (IO*E*RT) or low energy x-rays to the tumor bed while the surrounding healthy tissue is spared from radiation due to steep dose gradients [10]. This could lead to instant sterilization of the cavity surface from remaining tumor cells and delayed, or impaired, tumor cell proliferation between surgery and adjuvant therapies in deeper areas receiving lower doses. Furthermore, high single doses may elicit local (tumor bed) and systemic (immunogenic) responses which are not observed in this extent after conventionally fractionated radiotherapy [11, 12].

Several, mostly small retrospective single-institutional studies have shown efficacy of the approach (reviewed in [13]). However, those pioneering studies were using IOERT and thus faced classical technical challenges that come along with a forward-scattering irradiation system in a setting with cylindrical or spherical tumor cavities. Postoperative dose reconstructions from the Munster University group demonstrated that many patients receiving IOERT exhibited areas of inadequate coverage (due to inaccurately selected electron energies, inappropriate cone sizes or angle errors). As expected, they found that median and 2-year survival significantly improved with better coverage (MS: 15.2 with adequate coverage *vs*. 9.3 months with inadequate coverage; 2-year survival: 9.3% *vs*. 0%, p = 0.02) [14, 15].

In contrast to the previously used forward-directed electron beams, the isotropic low-energy X-ray irradiation source used in the TARGIT (TARGeted Intraoperative radioTherapy) trial for breast cancer (INTRABEAM system) [16] is specifically attractive in IORT for brain tumors, where post-resection cavities are normally of complex shape. IORT using such spherical applicators has been proven feasible in pediatric [17] and adult primary brain tumors [18, 19]. However, as there are no prospective data on safety and tolerability of IORT, we have designed INTRAGO, a phase I/II study built on the experience from past trials in several of which proof-of-principle has been demonstrated.

## Objectives

### Primary objectives

The primary goal is the definition of the maximal tolerated dose (MTD) of spherical isotropic kV-IORT in the setting of primary GBM.

### Secondary objectives

Secondary endpoints are progression-free (PFS) and overall survival (OS).

## Methods/Design

**Figure 1:**
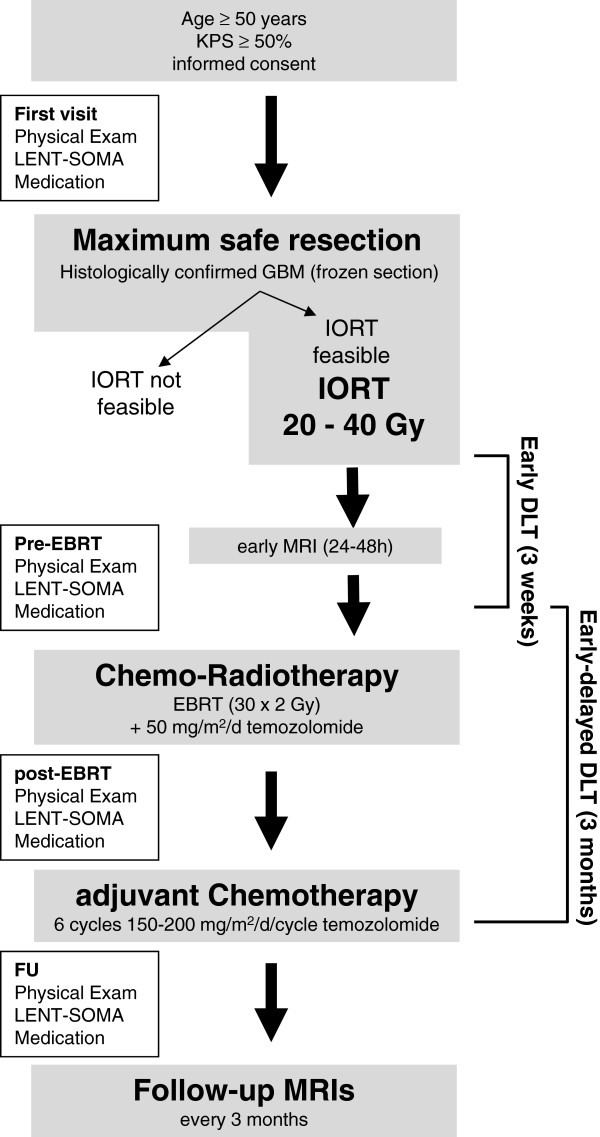
**Study Workflow.** Legend: KPS, Karnofsky Performance Status; GBM, glioblastoma multiforme; LENT-SOMA, Late Effects in Normal Tissues - Subjective, Objective, Management and Analytic (LENT-SOMA). IORT, intraoperative radiotherapy; EBRT, external beam radiotherapy; DLT, dose-limiting toxicity; MRI, magnetic resonance imaging.

INTRAGO resembles a prospective, open-label, single-arm dose-escalation study (Figure 1). Dose escalation will be conducted in a “classical” 3 + 3 manner with three patients entering each dose level. The decision to escalate to the next dose level is based on safety assessments after all patients of a cohort have reached month 3 (90 days) after IORT: If no dose-limiting toxicities (DLTs) occur in a cohort of 3 patients, the next cohort of 3 patients will be treated at the next higher dose level. If one of the 3 patients in a cohort experiences a DLT, an additional cohort of 3 patients will be treated at the same dose level: If no DLT occurs in the additional cohort, dose escalation will continue at the next higher dose level. Should one or more of the 3 patients in the additional cohort experience a DLT, the MTD is considered exceeded and dose escalation will stop. The preceding dose level will then be considered as the MTD. If two or all of the 3 patients in a cohort experience DLTs, the MTD will be considered exceeded and the preceding dose level will be defined as the MTD. If only a total of 3 patients were treated at the potential MTD level, the potential MTD needs to be confirmed by recruiting an additional cohort of 3 patients: If none or one of the 6 patients experience a DLT, the MTD will be considered confirmed. If two or more patients experience a DLT, the next lower dose level will become the potential MTD. If no DLT occurs at all, the highest dose level will be defined as the MTD. Thus, the MTD is defined as the highest dose at which one or no DLT will have been observed among 6 patients.

## Patient selection

Patients aged 50 years or older with a Karnofsky performance status (KPS) of at least 50% and a histologically confirmed (frozen sections) supratentorial unifocal GBM are included. The tumor location should allow maximum safe resection. However, patients with tumors that are likely to be only partially removable are also eligible for the study.

### Inclusion criteria

- Histologically confirmed glioblastoma multiforme in frozen sections
- Age ≥50 years
- Karnofsky Performance Index ≥ 50%
- Written Informed consent
- Adequate birth control (e.g., oral contraceptives)

### Exclusion criteria

- Astrocytoma ≤ WHO grade III
- Gliomatosis cerebri
- Multifocal lesions
- Infratentorial localization
- Previous cranial radiation therapy (any location)
- Uncontrolled intercurrent illnesses including, but not limited to, ongoing or active infection or psychiatric illness/social situations that would limit compliance with study requirements.
- Contraindications for general anaesthesia
- Bleeding or clotting disorders
- Contraindications for MRI or CT scans
- Pregnant or breastfeeding women

## Assessment of the primary objective (Safety)

The primary goal is to determine the MTD, which will be assessed on the basis of pre-defined dose-limiting toxicities (DLT).

Two types of DLT are defined:

1. Early DLTs (≤3 weeks after IORT):

- wound infections/wound healing difficulties requiring surgical intervention
- IORT-related cerebral bleeding or ischemia

2) Early-delayed DLTs (≤3 months after IORT):

- Symptomatic brain necrosis requiring surgical intervention
- Early termination of EBRT (before the envisaged dose of 60 Gy) due to radiotoxicity

DLTs will be assessed on the basis of clinical presentation (physical examination, KPS, current medication), imaging studies (MRI) and on the basis of a neurological assessment using the *Late Effects in Normal Tissues Subjective, Objective, Management and Analytic* (LENT-SOMA) scales defined by the EORTC/RTOG [20, 21].

### Evaluation of DLT: clinical exams, medication

Each follow-up visit has to include the most recent medical history, an inspection of the wound/scar and a thorough clinical exam. Episodes of partial or complex seizures must be documented. Wound healing (and the scar at FU) is followed with photo documentation. When performing a physical exam, there should be a specific emphasis on neurological functions. Specific awareness is advised for signs of cerebral edema (for example alterations in the level of consciousness, bradycardia, high blood pressure or inequality of pupillary size). All current medication and all changes made in the medication schedule have to be documented. Detailed information on doses (and dose changes) has to be only documented for corticosteroids and anticonvulsants.

### Evaluation of DLT: MRI

Each follow-up visit includes MRI with contrast-enhanced (gadolinium) T1, axial T2 and axial T2-FLAIR sequences. Ischemic areas can be delineated using perfusion diffusion-weighted imaging. It is challenging to distinguish early post-treatment blood–brain barrier permeability impairment from tumor recurrence and “true” brain necrosis. Here, methods such as *Lesion Quotient* (LQ), which resembles a ratio of the area delineated in a T2 sequence and the area of the corresponding contrast-enhanced T1 sequence may be applied [22]. In case MRI scans are inconclusive, positron emission tomography with amino acid tracers (such as ^18^F-fluoro-ethyl-tyrosine) can be offered as preferential modality.

## Management of DLT

Wound infections must be adequately treated, e.g. with dry absorbent dressing and, in case of positive wound swabs, systemic antibiotics should be administered matching to the antibiogram. In all cases of wound infection, blood workup (Complete blood counts, white blood counts, CRP) and imaging studies should be performed (CT or MRI) to rule out intracranial abscesses. Each case of wound infection (our healing difficulty) where conservative therapy fails and surgical revision is required is defined as DLT.

Due to the highly flexible positioning system of the IORT device, cases of cerebral ischemia or bleeding induced by the applicator are unexpected. Nevertheless, both were included into the protocol as DLTs and cases where (venous or arterial) ischemia or intracranial haemorrhage occur as a consequence of the IORT procedure (addition to the baseline risk), adequate therapy is required. Intracranial haemorrhages must be generally surgically removed if mass effects are exceeding the primary lesion volume. There is no symptomatic therapy for arterial or venous ischemia post resection. However, diffusion-weighted imaging (DWI) should be performed to document these events during follow-ups.

If radiation necrosis is suspected in MRI scans and no correlating clinical deterioration is noted, symptomatic therapy may not be necessary and observation is appropriate. For patients with mass effects or neurological symptoms, treatment options include conservative therapy with corticosteroids or anti-angiogenic substances (e.g., bevacizumab) or surgical debulking of the necrotic areas. The decision for either therapy should be made in interdisciplinary consensus (e.g., in interdisciplinary tumor boards). If surgery is required, the corresponding case will be defined as DLT.

All patients will undergo radiochemotherapy and will present five times per week at the linear accelerators. Complete blood counts (CBC), a chemistry panel, liver function tests (LFT) as well as renal function tests (RFT) are regularly performed to screen for hematotoxicity, hepatotoxicity or renal toxicity under temozolomide chemotherapy. Upon intolerance, incompliance or deterioration of CBCs, LFTs or RFTs, the therapy with temozolomide can be discontinued at any time point by any physician in charge. Any discontinuation of chemotherapy for more than 5 consecutive days has to be documented.

Cranial irradiation can elicit acute (side) effects occurring during treatment or early-delayed effects that appear within three months after radiochemotherapy [23]. In most cases, both acute and early delayed side effects largely resemble symptoms of mass effects (e.g., headache, nausea, worsening neurological symptoms), they are responsive to corticosteroids and they either gradually decrease in severity during daily radiotherapy (acute effects) or spontaneously resolve under cortisol (early-delayed). However, any case of radiotherapy-associated symptom deterioration that requires pausing of radiotherapy for more than 5 consecutive days has to be documented. If radiotherapy is entirely discontinued due to radiotherapy-associated side effects before the total dose of 60 Gy is reached, the case is considered to exhibit a DLT. As for chemotherapy, EBRT may be discontinued by any physician in charge.

## Assessment of secondary end points (efficacy)

Secondary end points are progression-free (PFS) and overall survival (OS). PFS is defined as the interval (in days or months) from IORT to the date of first detection of progressive disease according to updated RANO criteria [24] or the date of last follow-up. The RANO working group recommended tight restrictions for evaluating progression within 12 weeks after radiotherapy as irradiation transiently increases the permeability of the (peri)tumoral vasculature which in turn impairs the validity of MRI scans during this period [25].

In INTRAGO, only cases where new T1-enhancing lesions are detected *beyond* the 80% isodose or new lesions that are histologically proven to be new manifestations of GBM are defined as true progression within a timeframe of 12 weeks after EBRT.

After this period, progressive disease is defined if one of the following RANO criteria applies:

- New T1-contrast-enhancing lesions outside of radiation field on decreasing, stable, or increasing doses of corticosteroids
- Increase by ≥ 25% in the sum of the products of perpendicular diameters between the first post-radiotherapy scan, or a subsequent scan with smaller tumor size, and the scan at 12 weeks or later on stable or increasing doses of corticosteroids.
- Clinical deterioration attributable to tumor progress (and not to concurrent medication or diseases)
- Increased T2/FLAIR compared with baseline scan or best response after initiation of therapy on stable or increasing doses of corticosteroids.

OS is defined as the interval (in days or months) from IORT to the date of death from any cause.

## Imaging, interventions, follow-up

### First visit

Patients that are eligible for the study will be initially examined and the general medical condition (height, weight, general condition, Karnofsky index, medical history, medication) and the neurological (baseline) status (neurological exam and LENT-SOMA scales) will be assessed. All patients have to be willing and able to undergo repetitive MRI scans. Written informed consent must be obtained at least 24 h prior to surgery and IORT.

### Preceding imaging studies

All patients enrolled will receive preoperative MRI imaging (minimum 1.5 T) including contrast-enhanced T1-weighted magnetization-prepared rapid gradient echo (MP-RAGE) sequences to provide a 3D data set for image-guided surgery. Advanced imaging (such as perfusion or diffusion weighted imaging) may be performed at this stage but is not mandatory.

### IORT planning, risk structures

The optic nerve(s) (or the chiasm, respectively) and the brain stem are defined as risk structures and have to be identified on pre-operative scans. If any risk structure is located ≤2 cm to a T1-enhancing lesion in pre-operative MRIs, intraoperative imaging (e.g. intraoperative ultrasound or in-room CT/MRI) should be used after removal of the tumor (to account for potential brain shifts) to document distances to the applicator surface and to allow dose definition.

### Surgery

The resection procedure should be performed as image-(neuronavigation) guided surgery with techniques that meet individual center standards and preferences. Resection techniques may include suction, bi-/monopolar cautery or ultrasound aspiration. Intraoperative application of 5-aminolevulinic acid (5-ALA) can be used to determine residual tumor tissue. A maximum safe resection approach is recommended, but not mandatory. Due to the possibility of liquor accumulation (or retention) around the applicator and subsequent lowering of doses to the target volume (i.e. the resection cavity wall), ventricular opening during surgery should be avoided whenever possible.

### Frozen section

To establish the diagnosis of GBM, representative tissue samples have to be sent for histopathological examination. The frozen section/cryosection procedure can be performed according to local standards. In case histopathological hallmarks of grade 4 are present, the patient fulfils all inclusion criteria. If the diagnosis of GBM can not be reliably established with cryosection or if additional analyses are necessary, IORT must be omitted.

### Dose prescription and application of IORT

Following establishment of the diagnosis via frozen section and after the surgeon defines the macroscopic (or 5-ALA-delineated) tumor to be satisfactorily removed, IORT will be prepared. All potentially involved risk structures that have been defined in pre-operative imaging (see above) may have displaced consequent to neuro-shifting (i.e. reduced intracerebral pressure and loss of liquor after resection) and should be re-identified with intraoperative imaging (ultrasound or in-room CT/MRI).

For the two risk structures (Optical nerve/chiasm and brain stem), dose constraints of 12 Gy (Optical nerve) and 12.5 Gy (brain stem) are commonly accepted in LINAC-based EBRT according to the QUANTEC (*Quantitative Analyses of Normal Tissue Effects in the Clinic*) recommendations [26]. Since kV-irradiation shows an increased relative biological effectiveness (RBE) [27], adapted dose constraints (D_Max_) of 10 Gy apply for both structures during IORT with the (kV-based) INTRABEAM-System. D_Max_ to these structures are then defined intraoperatively on the basis of the dose-depth profiles of the corresponding applicator. If the doses to the risk structures at IORT (D_IORT_) exceed 10 Gy, IORT is technically not feasible and has to be omitted (screening failure). If any risk structure is likely to receive a D_IORT_ of > 10 Gy (e.g., the risk structure has direct contact to the applicator surface) it must be sufficiently shielded with cut-to-size tungsten-filed silicone shielding strips. Shielding with one layer tungsten-silicone strips will reduce the D_IORT_ by 90%.

Based on the cavity geometry and adjacent functional brain areas, the most suitable applicator will be chosen by the team of surgeons and radiation oncologists (sizes rage from 1.5- 5.0 cm in 0.5 cm steps). The applicator will be inserted in the cavity correct positioning and adjacent risk structures will then be again visualized using intraoperative imaging (ultrasound or in-room CT/MRI).

Fluids surrounding the surface should be ruled out or removed. The applicator is then taken out and mounted onto the INTRABEAM system. Next, the arm and the source are covered with the sterile drape and the mounted applicator is again fitted into the resection cavity. Radiation will then be initiated by a radiation oncologist for a defined time span as calculated by the machine software.

After IORT, surgery will be continued in a regular fashion without specific additional requirements.

### Radiation protection issues

IORT has to be delivered in accordance with federal, state and/or local regulations on radiation protection. IORT with the INTRABEAM® System does not require structural alterations if the operating room is approved for C-arm fluoroscopy [28].

### Early postoperative MRI

Early postoperative MRIs must be performed within a window of 24–48 h after surgery and must be analyzed in a standardized assessment. Before contrast application T1-hyperintense lesions must be used to evaluate residual blood/heme and T2-TSE, T2-FLAIR, and DWI to evaluate ischemia. After contrast application, T1-hyperintense masses or nodules (residual tumor tissue) have to be quantified and the following has to be documented:

- Complete resection: removal of at least 98% of the T1-enhancing lesions.
- Subtotal resection: removal of 88-98% of the T1-enhancing lesions.
- Partial resection: removal of less than 88% of the T1-enhancing lesions.

### Pre-EBRT visit

Before EBRT and concomitant chemotherapy is initiated, all patients will be re-examined (including a neurological exam, an update on medication and a re-assessment based on LENT-SOMA scales) to document changes with regard to the preoperative condition and to exclude contraindications for EBRT (such as impaired wound healing or active infection). Wound healing must be followed with photo documentation.

### External-beam radiotherapy (EBRT) treatment planning

Contrast-enhanced T1- and T2-FLAIR sequences have to be co-registered with the planning CT scan for radiotherapy planning. The planning target volume (PTV) is defined as the peritumoral edema in FLAIR sequences plus a 2 cm margin. Lenses, retinae, optical nerves and/or chiasm and brain stem have to be defined as organs at risk (OAR). The corresponding planning risk volumes (PRV) are defined as OAR plus a 3 mm safety margin. For all OAR, point dose (dose to a volume > 0.03 cm^3^) constraints apply (Table 1). In case an OAR was pre-irradiated during IORT (with or without shielding), the dose applied to the OAR during IORT (D_IORT_) has to be converted into an dose that is equivalent to a 2 Gy fractionation scheme (EQD2) using an α/β ratio of 2 and considering an RBE of 1.5 as follows (Table 2 gives exemplary D_IORT_ and EQD2 values):

**Table 1:** **Point dose constraints (dose to a volume > 0.03 cm** ^**3**^ **) for OAR**

Risk organ	Maximum point dose (EQD2)
Lenses	7 Gy
Retinae	50 Gy
Optical nerves/chiasm	55 Gy
Brain stem	66 Gy

**Table 2:** **Exemplary EQD2 values for various IORT doses (D**_**IORT**_ **) to risk organs**

D_IORT_	EQD2
3	6 Gy
4	9 Gy
5	13 Gy
6	18 Gy
7	24 Gy



Adequate target volume coverage should be considered by ensuring that 95-107% of the prescribed dose is received by 95% of the PTV. Both, 3D-CRT or IMRT may be applied, beam energies must at least be 6 MV. A total dose of 60 Gy delivered in 30 fractions (2 Gy/fraction) will be applied to the PTV. In case the PTV includes a PRV which would in consequence not receive a dose within the given constraints, a specific risk-organ PTV (PTV-R) has to be delineated to allow separate dose reconstruction.

### EBRT

EBRT must be initiated within 4 weeks after surgery and IORT. All patients will receive five fractions of radiotherapy per week on a conventional linear accelerator (LINAC) with thermoplastic mask fixation. Pausing of radiotherapy is allowed for maximum 5 consecutive days. During EBRT, patients are routinely seen once to twice weekly by physicians and evaluated for adverse reactions.

### Concomitant chemotherapy

During EBRT, all patients will receive concomitant temozolomide-based chemotherapy with a total oral dose of 50 mg/m^2^/d five times per week (on radiotherapy treatment days). Chemotherapy will be initiated at the first day of radiotherapy and will be continued until the last day of radiotherapy. Discontinuation of chemotherapy is allowed for maximum 5 consecutive days.

### Adjuvant chemotherapy

Four weeks after radiochemotherapy, adjuvant chemotherapy with temozolomide is initiated if the patients do not exhibit contraindications (low RBC, WBC and/or platelet counts, previous adverse reactions). The doses will be selected in accordance with the Stupp-protocol (150–200 mg/m^2^/d temozolomide, day 1–5 every 28 days) [2]. At least six cycles of chemotherapy should be applied. If a patient tolerates the cycling chemotherapy with temozolomide well, additional cycles may be applied.

### Post-EBRT follow-ups

Follow-up visits after radiochemotherapy will be scheduled quarterly. Each visit is preceded by brain MRI imaging studies (contrast-enhanced T1, T2 and T2-FLAIR sequences). Advanced imaging (diffusion- and perfusion-weighted sequences) is optional. The MRI scans are evaluated by a board-certified radiologist or neuroradiologist in accordance to updated RANO criteria (see above). Follow-up visits also include an assessment of the general medical condition (height, weight, general condition, Karnofsky index, medical history, medication) and the neurological status (neurological exam and LENT-SOMA scales).

## Serious adverse events

Treatment-emergent (i.e. IORT-related) events that are fatal, life threatening or classified as LENT-SOMA grade IV toxicity resemble a serious adverse event (SAE) and have to be documented. In case of death an autopsy will be pursued.

## Ethical aspects, trial registration

INTRAGO is approved by the local ethics committee (Medical Ethics Commission II of the Faculty of Medicine Mannheim, University of Heidelberg; 2013-548S-MA) and the Federal Office of Radiation Protection (Z 5-22462/2-2013-063). The trial is registered with clinicaltrials.gov, number: NCT02104882.

## Discussion

We here present the first dose-finding study on low-energy intraoperative radiotherapy for glioblastoma multiforme. Previous approaches used forward-directed electron beams that only inconsistently provided sufficient target volume coverage, leading to inconsistency in reported outcomes [15, 29–37]. Within INTRAGO, the spherically irradiating devices of the INTRABEAM system are used to enable geometry-optimized IORT. This may for the first time enable sufficient dose delivery to the resection cavity and to remaining tumor cells.

On may argue that there is no benefit of further dose escalation after resection due to failure of trials involving dose escalation or additional radiosurgery to the tumor bed [38]. We challenge this conclusion on the basis of two facts that involve the (crucial) time between surgery and adjuvant therapy:

First, it is known that the mean doubling time of a GBM stem cell may be as fast as 24 hours and consequently, the waiting time for EBRT correlates with overall survival [39, 40]. IORT is embedded in the surgical removal of the mass and thus will likely prevent and/or slow down this exceptionally fast tumor growth in this timeframe. Second, GBM growth shows dose-dependency as early studies showed that doses of at least 50 Gy are required to improve overall survival [41, 42]. In the RTOG 98–03 trial, patients treated with 66 Gy showed the worst (11.6 months) and those receiving 84 Gy showed the best survival rates (19.3 months) without increased rates of toxicities [43]. Of note, this study was conducted with rather “old” techniques and in the era of more advanced functional imaging (such as PET) and irradiation techniques (intensity-modulated radiotherapy) dose-escalation may be even more safely and efficiently conducted [44]. We think that the reason why several other dose-escalation trials were failing is likely to be related to processes occurring during the time between surgery and adjuvant therapy: any wounded site creates a specific stimulatory environment to promote healing, which inadvertently provides remaining cancer cells with strong pro-proliferative, pro-migratory and anti-apoptotic stimuli [45, 46]. In breast cancer, it has been shown that this unwanted response of the injured microenvironment can be attenuated with IORT [47]. This had direct consequences on the clinical outcome: patients that received IORT in a sequential operation, i.e. after the first surgery confirmed the diagnosis (‘post-pathology’ cohort) showed higher local recurrence rates (5.4%) compared with patients that received IORT during first surgery (2.1%; ‘pre-pathology’ cohort) [16]. Whether or not traumatic brain injury has a similar strong influence on GBM cell proliferation post surgery as observed in breast cancer has not been demonstrated yet. However, as the injured brain is very well known to respond with an impressive cytokine cocktail that efficiently promotes astrocytic activation and proliferation [48–50], we believe that similar (and, regarding survival, likely highly beneficial) quenching of the tumor microenvironment may be achievable after IORT for GBM.

Symptomatic brain necrosis requiring surgical intervention was defined as DLT. Brain necroses appeared in multiple IOERT trials and they mostly correlated with improved survival [29]. This, together with the fact that bevacizumab is a novel and effective option to conservatively treat brain necroses [51] was prompting us to only consider brain necroses as a DLT if they become symptomatic and if they require surgery. However, although we do expect cases of brain necrosis, highly elevated rates are less likely as it is well known that the irradiated volume of brain is the key determinant for this side effect [52]. The device used for IORT in INTRAGO uses low-energy (kV) photons that show exponential attenuation along their path [53]. Logically, the area receiving high(est) doses is a margin of maximum 1–1.5 cm width around the tumor cavity (IORT with 40 Gy surface dose at dose level III would result in doses of 12 Gy at 1 cm, 8 Gy at 1.5 cm and 4 Gy in 2 cm depth), which is a volume that is eventually not large enough to become clinical apparent.

INTRAGO is the first prospective IORT study in the era of temozolomide. The alkylating agent has become a crucial part of standard treatment after the pivotal EORTC/NCIC study showed a considerable improvement of both overall and long-term survival rates if the substance is added to radiotherapy and given as adjuvant chemotherapy [1]. It is believed that the increased rates of blood–brain barrier permeability impairments that are seen after radiochemotherapy (and which are often misinterpreted as progressive disease) are predominantly caused by temozolomide [25, 54]. This abnormal local reactions together with pre-clinical investigations point to additive and/or even synergistic activity of both modalities and it will be of specific interest to see whether IORT can further modulate these interactions [55, 56].

In conclusion, INTRAGO is the first dose-finding study on low-kV-IORT for newly diagnosed GBM in the temozolomide era with optimized geometry adaptation. It should provide a robust basis for subsequent randomized (phase II or III) trials, in which superiority over standard treatment must be tested.

## Abbreviations

3D-CRT: Three-Dimensional Conformal Radiation Therapy
CBC: Complete Blood Count
CT: Computer Tomography
D_IORT_: Dose applied to an OAR during IORT
DLT: Dose-Limiting Toxicity
DWI: Diffusion-Weighted (Magnetic Resonance) Imaging
EBRT: External Beam Radiotherapy
EORTC: European Organisation for Research and Treatment of Cancer
EQD2: Equivalent Dose in 2 Gy Fractions
FLAIR: Fluid Attenuated Inversion Recovery
FET: ^18^ F-fluoro-Ethyl-Tyrosine
FU: Follow-Up
GBM: Glioblastoma Multiforme
IMRT: Intensity-Modulated Radiation Therapy
INTRAGO: Intraoperative Radiotherapy in Glioblastoma Multiforme
IOERT: Intraoperative Electron Radiotherapy
IORT: Intraoperative Radiotherapy
KPS: Karnofsky Performance Status
kV: Kilovoltage (10^3^ V)
LENT-SOMA: Late Effects in Normal Tissues - Subjective, Objective, Management and Analytic
LINAC: Linear Accelerator
LFT: Liver Function Tests
LQ: Lesion Quotient
MP-RAGE: Magnetization-Prepared Rapid Gradient Echo
MRI: Magnetic Resonance Imaging
MTD: Maximum Tolerated Dose
OAR: Organ at Risk
OS: Overall Survival
PET: Positron Emission Tomography
PFS: Progression-Free Survival
PTV: Planning Target Volume
QUANTEC: Quantitative Analyses of Normal Tissue Effects in the Clinic
RANO: Response Assessment in Neuro-Oncology
RBE: Relative Biological Effectiveness
RFT: Renal Function Tests
RTOG: Radiation Therapy Oncology Group
SAE: Serious Adverse Event
TARGIT: Targeted Intraoperative Radiotherapy (randomized phase III study)
WBC: White Blood Cell Count.
